# Annual Meeting of the Virginia State Dental Association

**Published:** 1878-02

**Authors:** J. B. Hodgkin


### ARTICLE II.

### *Annual Meeting of the Virginia State Dental Association.*

(Continued.)

[Reported for this Journal by Prof. J. B. Hodgkin.]

Prof. Hodgkin said that he hailed with joy any plan of filling roots which promised better success than the methods commonly practised. For his own part he found it the most difficult of all the operations he was called on to perform, and he had to confess that no method he had used seemed perfectly satisfactory; and all were difficult of performance. So far as filling roots with cotton and os-artificial, or Hill's stopping, he had never felt satisfied with the results, or at least never felt satisfied that what he wished done had been done. There seemed to be no way of determining whether or not these materials had filled the root or not. The difficulty in the description of the method of Dr. Moore, as given by Dr. Johnston, was that an ideal root was filled, and not a sure-enough one, as the children say. The anterior superior roots were comparatively easily filled by any method; the ones that gave the real bother were the flattened roots of molars and bicuspids—those wretched lower molar roots, which seem as if designed purposely to baffle us.

To that prince of dentists, Dr. Edward Maynard, he was indebted for a method of enlarging canals—and he, in opposition to the practise of Dr. Johnston often found this necessary—which greatly simplified this work. A drill should never be used to enlarge a canal; you know where it goes in but you do not know where it will come out. He had seen a forcible illustration of this in the case of a gentleman who had a tooth filled and the roots enlarged by one of the most widely known dentists of the North. The gentleman for whom the tooth was filled came to Dr. H. with it aching terribly, and badly abscessed. It was a superior 3d molar. In his pocket-book he had a card which the operator had given him, on which was a diagram of the tooth and the filling, showing the three roots, filled with gold, and on that gutta-percha, the crown cavity being filled with gold. All very pretty on the card. But on removing the tooth, instead of three roots, there was but one, curved backward like a cow's horn, and the drill which had been used to enlarge the canal, had passed out into the alveolus, and the gold filling had followed it, protruding out of this cavity and pressing on the bone, with of course the inevitable result.

A piece of piano wire about the size of a small knitting needle and two inches long, made the very best instrument for enlarging root canals. Put this in a light wooden handle, and wood is best because of its lightness—touch has to take the place of sight here,—the end to be filed down to such a size as will pass into the canal, and made smooth with emery paper and a burnisher, and with a series of barbs cut on one side with a chisel, and we have here something that is simply a miniature mouse-tail file, and which will remove the soft dentine of the pulp canal very rapidly. These little instruments are very elastic and at the same time sufficiently hard, and when the barbs become dulled they can be burnished down and others cut on another side of the same wire. In this way in a few minutes a root can be sufficiently enlarged to get in a gold filling, and the same instrument answers for a plugger, with a little alteration.

Dr. Thackston said that at this late hour he would not attempt any prolonged discussion of the paper of Dr. Thompson. He could give no good explanation of the cause of the nervous exhaustion produced in us by certain patients. That the question of brain worry or brain fag had something to do with it, it was undoubtedly true, but still it seemed to require other explanations, and was one of those wonderful psycological facts incapable of explanation. Sometimes we feel better on approaching than on receding from a patient, but what this influence is he could not tell. That it is a fact, is beyond question, but we cannot say how or what it is.

In reference to Riggs' disease, so called, which Dr. Thompson had spoken of in his paper, it may not be known to the younger members of the profession, but this subject had been investigated in the most careful and exhaustive manner by Dr. Hayden, thirty five years ago, and was at that time a subject of the most bitter controversy, and was designated by Dr. Hayden, as "conjoined suppuration of the gums and alveolar processes," and the conclusion was reached that after the disease had reached a certain stage there was no remedy. His own experience as a dentist had led him to the same conclusion, and he had found that after all his labors to retain such teeth, the termination of the disease was, as a rule, extraction. The rough calcareous deposit on the teeth down to the apices of the roots was impossible to remove with any instruments, and it consisted of exceedingly hard pointed, corundum-like crystals, sharp as barbs, often deposited to the end of the root. He did not consider this to be tartar. All this parade about Riggs' disease is a sham—the disease was well and accurately described by Dr. Hayden, 35 years ago. Nor did he believe, as Dr. Atkinson teaches, that the alveolar border would or could be reproduced after removal.

Prof. Hodgkin related an interesting account of a boy aged about 10 years, whose lower maxilla from the right angle to the left articulation was removed entire. The bone was extensively necrosed, the result of profound mercurial salivation. The operation was one of great neatness, the entire bone being removed from within the mouth, thus avoiding hemorrhage and disfigurement of the face. The extraordinary part of the affair was that the entire jaw had been reproduced in the space of about two years—smaller it is true, but as perfect in form and function as the original bone. The right lower second molar was in position, but no other teeth. It is interesting to know that nature from the periosteum can thus reproduce an entire bone; it would be still more interesting could the teeth be reproduced. The articulation of the new jaw is perfect, so far as can be discovered.

Dr. Thackston related a case somewhat similar, in which the bone was destroyed by phosphorous poisoning, and reproduced, smaller and less perfect than the original. He did not think, however, that any one had been able to reproduce the alveolar process when it was once destroyed.

While he was speaking he would advert to a subject belonging to the scope of the paper. He referred to the statements lately made by sundry gentlemen in societies, in which they asserted that a great deal of unnecessary time was spent over fillings, and that the time of performing operations (gold fillings) need not average over fifteen minutes, and strangely enough such statements were endorsed by such men as Dr. Atkinson. He wished to warn the young men against falling into such errors.

Prof. Hodgkin stated that he had it from a friend that another prominent New York dentist stated that he had filled twenty-five cavities with gold in two hours.

Dr. Moore said that he had seen some of the work of some of these prodigious men, and it certainly bore evidence in point of worthlessness of having only taken a short while.

Prof. Hodgkin suggested that happily the reason the fillings come out, might be found in an excuse for such tumblings at the hands of a man more ready at making excuses than he was for enduring work;--the excuse he gave, was that some teeth had no affinity for fillings, and in such teeth they would not stay.

Adjourned to meet at four P. M.

### Second Day—Afternoon.

The subject of Operative Dentistry was continued by Dr. Chewning, of Fredericksburg, who spoke of his unsatisfactory efforts at bleaching teeth. In many cases in which the bleaching seemed perfect, in a few weeks or months the discoloration returned. These failures were mortifying, and any light on the subject would be thankfully received. He had used chlorinated soda, usually

Dr. Johnston's method, and success, was much the same as that of the previous speaker. He was not often successful. The trouble seemed to be that the tubules of the dentine were filled with decomposed blood, and no agent that would not displace this, would accomplish the desired result. The worst cases were those in which the teeth had been devitalized by a blow or fall. He had used with good results dry chloride of lime, and sometimes by lining the tooth with Gillois' cement, the dark line was taken away by the whit back-ground.

Prof. Hodgkin agreed with the previous speaker, that these were among the most uncertain operations we were called on to perform. In spite of his most patient efforts, the case occasionally relapsed into its original objectionable condition. Had used chlorine gas with great transient benefit, but it seemed no more permanent in its effect than the ordinary bleaching agents, which mostly depended on the disengagement of their feebly combined chlorine. The use of acids was recommended by some, but it seemed that any acid which would bleach the tooth would injure its structure.

Dr. Thackston said his thirty years' experience was about equivalent to that of the previous speaker, and was in the main unsatisfactory. The discoloration was due to the remains of the circulation left in the tooth structure, and our success depended in part on the difference in the teeth operated on. In open, porous teeth, the operation of bleaching was speedily performed, and as speedily failed; in the denser and more solid teeth, the progress of restoration was slow, but the results were more satisfactory. The soonest bleached, the soonest relapsed, was his observation. He had tried everything that had been spoken of from time to time—oxalic acid, &c., but he had no certain success.

Prof. Hodgkin gave some account of Bonwill's method of pivoting teeth, illustrating his remarks by diagrams on the blackboard. He spoke of this method as the best, in his judgment, yet devised for pivoting, and earnestly advised the use of this or some other method of inserting artificial teeth in preference to plates.

Dr. J. B. Wood, of Richmond, gave an interesting account of a method of pivoting, practised by him, which had proven so satisfactory as to entirely displace all other pivots. His plan was to get his jeweller to draw a soldered tube over a piece of half round gold wire, which had been folded on itself, thus making a split pivot within a gold tube, the one fitting the other perfectly. The root was prepared much as for a wooden pivot, with the addition that near the orifice of the pulp chamber undercuts were made, and so much enlargement as would allow gold to be packed in around the tube. The tube is now placed in position, and fits neatly the enlarged canal, and cohesive gold is packed around the outside of the end and a flush filling made, the outside lower end of the tube being roughened to hold the gold more perfectly. No rubber dam is necessary. The most effective hœmostatic he knew of was simple tincture of iodine. It stopped the flow of blood and of all moisture more perfectly than anything he had ever seen.

The tube in position and the filling completed, the rest of the operation was simple. The wire was placed in position in the tube, the end a little projecting, a platinum disk was filled over this with a burnisher, a plate tooth was backed, and the relative positions of the two secured by an impression, the case soldered and finished. He had found this method to be easy and satisfactory. The wire and tube he had prepared in some little quantity, and it was always ready for use.

Dr. Wood's method for filling roots was different from that pursued by some who had spoken.* He used both Hill's stopping and common India rubber (vulcanizable gum), for filling roots, using very small fragments of Hill's stopping and jeweler's brooches warmed to force it up. In very small roots he used a fine silver instrument, coating it with rubber, and forcing it gradually up. In teeth experimented on in the hand, he had repeatedly, from too great pressure, seen the Hill's stopping exude from the apicial foramen.

In treating abscesses, he considered it essential to rapid cure that the foramen should be tightly stopped. He used tincture of iodine, pumping it in till it appeared in the

*Note.—Dr. Wood described these methods to the reporter *after* the close of the evening's session, but for the sake of convenience they are introduced here.

fistulous opening, and then stopped up the foramen as tightly as possible with floss silk, the idea being that any liquid accumulating in the root is more readily decomposed if in bulk, but if outside it is carried off by the absorbents. He has had uniform success by this method. He fills the root as soon as circumstances will justify.

Dr. Wood also reports 32 cases of extraction and replantation, with 8 failures. He considers it absolutely necessary to success that the tooth should be returned to the socket as soon as possible and to accomplish this he only fills the end of the root, at the time; filling the rest of the tooth after it has become firm. These cases represent incisiors, bicuspids and lower molars.

Dr. Burton described a method of pivioting a tooth which he thought possessed some merit. It consisted in filling the root—a badly decayed one—with ordinary red gutta-percha, and warming the wooden pivot, forcing this up into place. A tooth so pivoted, and subject to great and habitual strain, remained quite firm.

Prof. Hodgkin took exception to the metal tubes described by Dr. Wood, and metallic pivots of that class in general on the ground, that the slight motion inseparable from the act of mastication, would cause wear and consequent loosening of the pivot. The necessarily small size of the pivot and the great strain brought on the tooth will loosen it in a short time. The method of Bonwill has the advantage also of a fluid tight joint made by gutta-percha, while at the same time this latter material is not exposed to wear; while the tooth itself is held firmly in place by a bolt and nut.

The name of Dr. T. G. Cowardin was proposed for membership.

Dr. Steel, from the special committee on the President's address, offered the following report:

The committee, to whom was referred that portion of the President's address, advising the expediency of seeking legislative protection for our profession, beg leave to report that, having carefully considered the subject, and being satisfied that some action should be taken, respectfully recommend that the Association appoint a committee, representing different parts of our State, of whom our President (Dr. Thackston) shall be the chairman, to prepare a bill relative to this subject, to be presented if possible, to the present Legislature for their action.

Dr. Wood thought that the time had not yet come for this sort of action, we want better organization, and the position this resolution commits us to is not practicable. It would be a disastrous thing to go before the Legislature in this matter and fail; better than that, that we should not undertake it. The medical profession tried to get legislative protection and failed. We want better organization, we want the name and the support of every dentist in the State. We shculd get these dentists to see their legislators, and there is hardly time for this this winter.

Dr. Scribner. We have waited for years for organization and it has not come; it seems further off than ever. We had better go on now, for no good will come of waiting.

Dr. Perrow said that something like this was moved eight years ago, and it was said then that we must wait for the medical men. As for the legislators they had been talked with, a good many of them, and they were willing to do what we want, so that we do not involve the State in any expense. This is the best time now, as the Legislature will probably not meet again for two years, As the law stands now any one who takes out a license can practice.

The subject was discussed further by Drs. Thompson, Hodgkin and Wood, when the resolution was adopted and the Association adjourned till the next day.

### Third Day.—Morning.

The Association, Dr. Thackston in the chair, reassembled at half past ten A. M., and after the routine business, a report on Mechanical Dentistry was read by Dr. Perrow.

Dr. Moore made a verbal report, stating that there was nothing new to report, unless it be on the subject of the Soft Rubber Discs, which were so extensively advertised, and which he regarded as humbugs. He expressed the conviction that celluloid was a more agreeable base than vulcanite, but not so durable.

Dr. Chewning said he thought that most of the dentists if they were on good terms with the Goodyear Dental Vulcanite Company, would prefer rubber to celluloid.

Dr. Johnson remarked that, for himself, he was unfortunately in the clutches of Bacon, and as a consequence, had gone back to metallic bases. His patients were, however, no losers, but rather gainers by this, as he considered metal plates were much superior to vulcanite, &c., in point of comfort as well as durability. Whenever the patient could bear the expense, he always preferred gold. He had not used celluloid for two years, but all the pieces he had made were wearing well, and giving perfect satisfaction. He thought it stronger than rubber. He had never seen a plate split. All these bases, (non-metallic) were non-conductors of temperature, and so, on that account were objectionable. He thought these plates caused more absorbtion.

Dr. Chewning wished to know if a celluloid plate removed from the mouth some little time, fitted as well on being replaced.

Dr. Henkle replied that he had not experimented in that way, but he thought that all plates should be placed in water, when out of the mouth, which would prevent warping. He had worked celluloid as soon as he could obtain plates to work, and had had no larger proportion of pieces to replace than of vulcanite. Some of his pieces, he was sorry to say, had turned miserably dark. We cannot altogether depend on it. With regard to the suits which the Celluloid Company had promised to defend, they had failed to meet his case, and he had to pay up to the Goodyear men.

Dr. Scribner said that his practice was limited mostly to young people, and as a consequence he did but little mechanical work. He preferred gold as a base. His failures with celluloid were as numerous as his successes.

Dr. Keesee stated that he had found celluloid to answer nearly every necessary purpose, and he considered it as a success, with the slight exception that some of the plates turned dark in the mouth. He had worked it exclusively for three years. One of its faults seemed to be that the bite was in some unexplained way nearly always lengthened, so as to necessitate more or less subsequent grinding of the teeth to secure perfect articulation. He asked what was the objection to Weston's metal.

Dr. Sprinkle replied that Weston's metal would not last.

Dr. Henkle said that in at least one case he had found Weston's metal superior to either rubber or celluloid. It was a desirable base in some respects, and was easily worked. All materials were liable to break.

Dr. Scribner stated that he heard a good deal said about Weston's metal, and asked the question what it was. He thought the dental profession ought not to deal with those patented nostrums, as they were responsible to their patrons for what was used, and ought not to use them without they knew what they were made of.

Dr. Keesee asked if Dr. Scribner knew the exact ingredients of the amalgam he was using?

Dr. Thackston thought of all cheap, durable materials used, Weston's metal was the best. He had tried it, and would advise the members to do so, as it had answered the purposes claimed for it. He was under the impression that block-tin was one of its principal ingredients. It was a valuable base, and was doing admirable service. Of course it will tarnish somewhat in some mouths, but he had never seen a split plate. So far as the action of the fluids of the mouth are concerned, eighteen carat gold is acted upon, and in some cases entirely disintegrated. The secretions of some mouths will destroy almost anything. Silver was the least stable of all bases. He would decidedly prefer celluloid to vulcanite.

Dr. Thompson had been using celluloid now for some years, and was perfectly satisfied with its merits. In manipulating it, he advised the use of coarse plaster.

Dr. Henkel exhibited a plaster cast, where there was contraction and recession of the superior maxilla caused by the extraction of the superior cuspids, which came in an irregular position on the outside of the arch. The diagnosis was that instead of extraction, the arch should have been spread by mechanical appliances. so as to have allowed the cuspids to have been brought into their correct position, thereby preserving the beauty of the face and to be useful for mastication.

Dr. Chewning exhibited a mode showing the irregularity of teeth, described the nature and character of the case, and the mode of treatment. A full and clear history of the case, involving its difficulties, was also made to the Association. This case was considered one of unusual interest, and was carefully examined and fully discussed by the Association.

On motion of Dr. Henkle, the report of the Committee on Mechanical Dentistry was passed by.

Dr. Thompson moved that the rules be suspended so as to appoint a committee to frame a bill to be submitted to the Legislature, as provided for in the report of Dr. Steele. Adopted.

Drs. Thackston, Henkle, Moore, Sprinkel and Thompson, were appointed the committee. Adjourned.

### Third Day.—Afternoon.

Dr. T. G. Cowardin was elected to membership in the Association.

The committee appointed to draft a petition to the Legislature, made the following report:

A bill to regulate the practice of Dentistry, and to protect the people against empiricism in relation thereto in the Commonwealth of Virginia, and providing penalties for the violation of the same.

SECTION 1.—Be it enacted, etc,—That from and after the passage of this Act, it shall be unlawful for any person, except regularly authorized Physicians and Surgeons, to engage in the practice of Dentistry in the Commonwealth of Virginia, unless said person has graduated and received a Diploma from the Faculty of a reputable institution where this specialty is taught, and chartered under the authority of some one of the United States, or of a Foreign Government acknowledged as such, or shall have obtained a certificate from a Board of Examiners duly appointed and authorized by the provisions of this Act to issue such Certificate.

SEC. 2.—That the Board of examiners shall consist of six (6) practitioners of Dentistry, who are of acknowledged ability in the profession. Said Board shall be elected by the Virginia State Dental Association at their next annual meeting, as follows: Two shall be elected for one year, two for two years, and two for three years; and each year thereafter, two shall be elected to serve for three years, or, until their successors are elected. The said Board shall have power to fill all vacancies for unexpired terms, and they shall be responsible to said State Dental Association for their Acts.

SEC. 3.—That it shall be the duty of this Board:

*First.*—To meet annually at the time and place of meeting of the Virginia State Dental Association, and at such other time and place as the said Board shall agree upon, to conduct the examination of applicants. They shall also meet for the same purpose at the call of any four members of said Board, at such time and place as may be designated. Thirty days notice must be given of the meetings, by advertising in at least two of the daily papers published in the Commonwealth of Virginia.

*Second.*—To grant a certificate of ability to practice Dentistry, which certificate shall be signed by said Board, and stamped with a suitable seal, to all applicants who undergo a satisfactory examination, and who receive at least four affirmative votes.

*Third.*—To keep a book in which shall be registered the names, (and qualifications of such, as far as practicable,) of all persons who have been granted certificates of ability to practice Dentistry under the provisions of this Act.

Sec. 4.—That the book so kept shall be a book of record; and transcripts from it certified to by the officer who has it in keeping, with the seal of said Board of examiners, shall be evidence in any court of this Commonwealth.

Sec. 5.—That four members of this Board shall constitute a quorum for the transaction of business, and should a quorum not be present on any day appointed for their meeting, those present may adjourn from day to day until a quorum is present.

Sec. 6.—That any person who shall in violation of this Act, practice Dentistry in the Commonwealth of Virginia, shall be liable to indictment in the Circuit, County, or Corporation Courts, and on conviction shall be fined not less than fifty, or more than two hundred dollars; Provided, That any person so convicted shall not be entitled to any fee for services rendered, and if a fee shall have been paid, the patient, or his or her heirs may recover the same as debts of like amount are now recovered by law.

Sec. 7.—That all fines collected shall inure to the public school fund of the County or Corporation in which the prosecution occurs.

Sec. 8.—That nothing in this Act shall apply to persons who shall have been engaged in the continuous practice of Dentistry in this Commonwealth for three years or over, at the time of, or prior to the passage of this Act; but such practitioners shall be entitled to a certificate without examination by said Board.

Sec. 9.—That to provide a fund to carry out the provisions of the third section of this Act, it shall be the duty of the said Board of Examiners to collect from those who receive the Certificate to practise Dentistry, the sum of thirty (30) dollars each; of which sum, if there is any remaining after liquidating necessary expenses, the balance shall be paid into the treasury of the said Virginia State Dental Association and be kept as a fund for the more perfect carrying out of the provisions of this Act.

Dr. Johnston, from the Committee on Dental Literature, made a verbal report. He might have written one, but disliked writing. On motion the subject was passed, as was also the subject of Pathology, Dr. Steele having no report ready.

Dr. Wood gave an interesting account of the sufferings of a young lady from neuralgia, the diagnosis of which was obscure. A number of her teeth had been filled, and one of these was an amalgam filling in the proximal face of a molar. These fillings were all good, but he fancied that possibly the amalgam filling had something to do with it, and it was removed; but the agonizing pain, which had been persistent for eighteen months, still continued. Extraction of the molar developed the fact that calcification of the pulp was the cause of all the trouble.

Dr. Henkel related a similar experience with a tooth having a gold filling instead of amalgam.

Dr. Wood made an informal report from the Committee on Histology and Microscopy.

An essay by Prof. Hodgkin, on Life Assurance was read, and on motion the thanks of the Association tendered for the same.

The election of officers for the ensuing year being in order, the following gentlemen were elected:

*President.*—Dr. J. Hall Moore, of Richmond.

1*st Vice President.*—Dr. S. H. Henkle, of Staunton.

2*d Vice President.*—Dr. Geo. B. Steele, of Richmond.

3*d Vice President.*—Dr. Jno. W. Scribner, of Charlottsville.

*Treasurer.*—Dr. James F. Thompson, of Fredericksburg.

*Recording Secretary.*—Dr. George F. Keesee, of Richmond.

*Corresponding Secretary.*—Dr. L. M. Cowardin, of Richmond.

*Reporting Secretary.*—Dr. George H. Chewning, of Fredericksburg.

*Executive Committee.*—Drs. J. B. Wood and Jeter, of Richmond, and Dr. Frank L. Harris, of Harrisonburg.

After some remarks by a number of members,

Dr. Wood was appointed to conduct the president-elect to the chair, when the retiring president addressed him as follows:

Mr. President-elect,—I congratulate you upon the distinguished honor which the voice of this Association has just conferred. In making you its first and presiding officer the Association has vindicated its title to justice and judgment, and in thus honoring *you*, it has no less honored itself by illustrating its prudence and wisdom.

I take pleasure in investing you with the dignities, the privileges, and the responsibilities of your elevated position, knowing that I commit to safe and able hands the interests and objects of our organization, feeling the perfect assurance that you will be faithful to the trust and confidence reposed in you, and that you will be cheerfully aided and cordially sustained in all the duties and functions of the office to which you have been elected.

Receive, then, this badge of authority, and with it the expression of our conviction that in *your* hands it will be held "*evenly*" and wielded *worthily;* and that under your government, our Association will steadily move on to the accomplishment of its ends and objects, and to the realization of all its hopes and aims.

Dr. Moore, the newly-elected president, responded in appropriate terms, thanking the Association for the honor conferred upon him, and promising his best endeavors to promote the interest of the Association and the profession at large.

On motion of Dr. Steele, a resolution of thanks was unanimously tendered to the retiring president, (Dr. Thackston) for the faithful and efficient manner in which he discharged the duties of the office of president.

Dr. Thackston returned thanks for the compliment, after which the Association adjourned to meet on the Tuesday after the second Monday in December, 1878.

Immediately after the adjournment of the Association, the Executive Committee met and elected Dr. Jud. B. Wood chairman.

---

### ARTICLE III.

### *Jottings in Operative Dentistry.*

BY JAS. F. THOMPSON., D. D. S., FREDERICKSBURG, VA.

[Read before the Virginia State Dental Association, Dec. 12, 1877.]

